# Village Centres for the Disabled

**Published:** 1920-03-20

**Authors:** 


### Village Centres for the Disabled.

The Village Centres Council for the concurrent treatment and training of disabled ex-Service men, of which Lord Haig is president, is holding its "memory day" next Wednesday, and the enlightened methods of the organisation in repaying some of the debt owed to those who fought and suffered deserve the support of all who are not content with lip service only. The objects speak for themselves. They are to assist the State in accelerating the restoration to health and fitness for work of ex-Service men whose disablement can be remedied by methods of medical treatment and mental and physical re-education; to associate with the medical treatment such vocational training in the open air and in workshops, especially for such occupations as can be practised on a large country estate; to offer attractive and co-operative conditions of social life which will appeal to the men; to enable men with their families to occupy cottages and homes and a bit of land on fair terms during the curative period, which may in many cases be prolonged; to speed the men's recovery so that with the help of their State pensions and their own resources, and those put at their disposal by the Ministry of Labour, they may be able to return to their former homes and previous occupations, or earn a new livelihood in healthy rural trades or callings; to utilise the village centres permanently in the interests of disabled ex-Service men, by way of settlement and otherwise, and as a national establishment for the restoration to health and for the physical re-education of other persons injured in the service of the State.

The organisation has certainly been prompt, and the first village centre has made an excellent start at Enham Place, near Andover, purchased for £30,000. The experiences already gained here have been full of interest and encouragement; there were 150 men in residence as long ago as October last, and several had passed out as wage-earners, or fit to return to business, or sufficiently cured to continue training at non-medical centres. A great variety of training is combined with curative treatment, and the families of some of the men are in residence on the estate. It is a sensible and humane conclusion—which is set down by the Administrator and Medical Director—that the restoration to health of many men is actually retarded owing to the fact that their wives and families are living in distant parts of England, and some men are obliged to leave in order to be near their homes. The provision of cottages is one of the urgent needs of the centre for the accommodation of men (with their families) who, from their permanent disabilities, may never be able to compete on equal terms with their more fortunate fellows.

The first four cottages (a memorial gift) were in course of erection towards the end of last year. There is great scope for the development of this beneficent work begun on such sound lines, and it should be backed not only by a spasmodic flag-day gift, but by steady and regular help. It has the warm support of the various Government Departments concerned.

